# Preparing auditory task switching in a task with overlapping and non-overlapping response sets

**DOI:** 10.1007/s00426-023-01796-x

**Published:** 2023-02-15

**Authors:** Sophie Nolden, Iring Koch

**Affiliations:** 1 Cognitive and Experimental Psychology, Institute of Psychology, RWTH Aachen University, Aachen, Germany; 2 Department for Developmental Psychology, Institute of Psychology, Goethe-University Frankfurt, Theodor-W.-Adorno-Platz 6, 60323 Frankfurt am Main, Germany

**Keywords:** Task-switching, Audition, Response-set overlap, Preparation, Stimulus dimensions

## Abstract

We used a variant of cued auditory task switching to investigate task preparation and its relation to response-set overlap. Previous studies found increased interference with overlapping response sets across tasks relative to non-overlapping motor response sets. In the present experiments, participants classified either pitch or loudness of a simple tone as low or high, hence, both tasks were constructed around common underlying integrated semantic categories ranging from low to high. Manual responses overlapped in both category and modality for both tasks in Experiment 1A, whereas each task was related to a specific response category and response modality (manual vs. vocal) in Experiment 1B. Focusing on the manual responses in both experiments, the data showed that non-overlapping response sets (Experiment 1B) resulted in a decreased congruency effect, suggesting reduced response-based crosstalk and thus better task shielding, but at the same time switch costs were increased, suggesting less efficient switching between task sets. Moreover, varying preparation time (cue-stimulus interval, CSI) showed that long CSI led to better performance overall. Our results thus suggest that when non-overlapping response sets share common semantic categories across tasks, there is no general benefit over overlapping response sets.

## Introduction

Being faced with multiple tasks in rapid succession is very common in daily living and yet comes along with a range of challenges for our cognitive system. When switching between tasks, it is common to find so-called switch costs, i.e., performance impairments in response times (RTs) and/or error rates (Meiran, 1996; Rogers & Monsell, 1995; see also Allport et al., 1994; for a recent review, see Koch et al., 2018). As a simple example, imagine two tasks that need to be performed multiple times in an unpredictable sequence such as changing the bass and the volume of songs on a playlist with random order. Switching from the adjustment of the volume to the adjustment of the bass (or vice versa) would thus be slower and/or more error prone than doing the same task twice in a row. In a task-switching situation, each task comprises specific properties related to stimuli, responses, stimulus–response mappings, and many more. The extent to which performance suffers from task switching depends on the specific higher order representations of the cognitive and motor task requirements (i.e., task sets) of the two tasks and their overlap (see Kiesel et al., 2010). Importantly, task switch costs can usually be reduced when there is more time to prepare for a switch (e.g., Meiran, 1996, 2000; Rogers & Monsell, 1995; for reviews see Kiesel et al., 2010; Koch et al., 2018; Koch & Kiesel, 2022). The goal of the present paper was to investigate the role of task preparation in the specific context of response-set overlap which we will review in the following.

There is evidence in the literature that switch costs are greater with bivalent responses, that is when the same responses are part of two different task sets, than with univalent responses, that is when all task sets comprise unique responses (Meiran, 2000). For example, Meiran (2000) used two visual-spatial localization tasks requiring participants to categorize the location of a target stimulus on a 2 × 2 array on the screen as either left vs. right or as up vs. down. He found that switch costs were greater for manual responses when they were made with two fingers along a diagonal axis of a response panel (e.g., lower left key and upper right key), so that the responses overlapped for the two spatial localization tasks (bivalent responses), than when there was a separate response for each of the four locations (univalent responses; see also Brass et al., 2003).

Yeung and Monsell (2003) differentiated overlap in response modality (manual or vocal) and overlap in response category (digits or directions) and showed that response overlap increased switch costs in both cases. These findings suggest that non-overlapping responses can be beneficial in situations that require a high amount of switching between tasks, most likely due to easier switching between response sets that are substantially different, so that individual responses do not have competing associations with both tasks.

An important feature of most previous studies was that the stimuli were bivalent, too. For example, in Meiran’s (2000) paradigm, a stimulus located at the left upper part of the screen could be categorized as both “left” or “up”. It has been shown that switch costs, with overlapping responses, are smaller for univalent stimuli than for bivalent stimuli. As an example for univalent stimuli, the target for the left vs. right task was presented vertically in the middle, or the target for the up vs. down task was presented horizontally in the middle, so that the targets afford only one task instead of two tasks (Koch et al., 2003).

Importantly, bivalent stimuli can produce congruency effects. For example, for bivalent responses along the left-down to right-up diagonal, an upper left target stimulus would require a left response for the horizontal task but a right response for the vertical task. Many studies found that performance is worse for such incongruent targets than for congruent target stimuli (e.g., Rogers & Monsell, 1995; see Kiesel et al., 2010, for a review).

Based on these considerations of the effects of response set overlap and of congruency, Nolden and Koch, (2022) developed a cued auditory task-switching paradigm requiring pitch and loudness judgments on an integrated semantic category (i.e., low to high), so that the stimulus categories overlapped across the two dimensions. Note that, for example in Meiran’s, (2000) paradigm, there were four different stimulus categories (i.e., left, right, up, down). Participants were instructed with a visual cue about the to-be-performed task, then the tone was presented and participants gave their response. Using our setup with auditory stimulus dimensions that have common semantic categories, we varied response-set overlap (either manual responses only, or, in case of non-overlapping response sets, manual and vocal responses). In case of overlapping response sets, participants used the same manual responses for both tasks, but in case of non-overlapping response sets, the response modality (manual or vocal) differed across tasks. Yet, unlike previous studies, we did not find a beneficial effect of reduced response set overlap on switch costs (but a non-significant trend to the opposite pattern), suggesting that reduced response set overlap does not seem to reduce switch costs generally. Thus, the absence of the benefit of reduced response overlap across tasks could likely be due to the integrated underlying semantic category in this specific task, which produced particularly strong task interference at the level of stimulus categories and which may render response-based interference less critical.

Aside from response-set overlap, other task-set aspects play also a key role in task switching. Interference between tasks may arise when the currently irrelevant task is activated to a certain extent. In Nolden and Koch, (2022), stimuli were identical for both tasks (classifying pitch vs. loudness), and thus were bivalent, as opposed to univalent stimuli. Bivalent stimuli may lead to an activation of the relevant and the irrelevant task as well because the stimuli are part of both task sets (and effect termed “exogenous cuing of task set” by Rogers & Monsell, 1995; see also Rubin & Koch, 2006) and thus can lead to a congruency effect (see above). This refers to worse performance in an incongruent situation, for example, as in Nolden and Koch (2022), when a stimulus is high in volume and low in loudness, than in a congruent situation, for example when a stimulus is high in volume and high in loudness. In this study, incongruent trials comprised conflicting stimulus–response (S-R) mappings in case of overlapping motor response sets, but also conflicting values of the two stimulus features, both for overlapping and non-overlapping motor response sets. We observed a congruency effect even when motor response sets did not overlap, but this effect was increased when there was response set overlap. This larger congruency effect is most likely due to the overlapping and conflicting S-R mappings with bivalent responses (Rogers & Monsell, 1995). Hence, with a common semantic category, response-set overlap seems to be related more strongly to the difficulty of keeping separate the two stimulus dimensions (see task shielding; Goschke, 2013) and less to the difficulty of switching itself.

The aim of the current study, using the cued auditory task switching developed by Nolden and Koch, (2022), was to examine the role of task preparation with overlapping vs. non-overlapping response sets. To this end, we varied preparation time by manipulating the cue-stimulus interval (CSI, see also Nolden & Koch, 2017, Seibold et al., 2019). When there is more time to prepare a task switch, switch costs can typically be reduced (e.g., Meiran, 1996) with some residual switch costs remaining even with ample preparation time (e.g., Meiran, 2000; Rogers & Monsell, 1995; for reviews see Kiesel et al., 2010; Koch et al., 2018). Specifically, with respect to our experimental setup, increased preparation time may reduce switch costs more in case of non-overlapping than overlapping motor response sets if preparation is used to isolate a subset of potential responses among all response alternatives.

We conducted two structurally very similar experiments, Experiment 1A with overlapping motor response sets (manual responses in both tasks) and Experiment 1B with non-overlapping motor response sets (manual responses in one task but vocal responses in the other task). The between-experiment comparison was the main focus of the analysis, to systematically investigate the role of task preparation on auditory task switching and its interaction with motor response set overlap. For this comparison, we focused on the manual responses, which were common (and thus strictly comparable) for both experiments.

We predicted increased or similar switch costs for non-overlapping motor response sets in comparison to overlapping motor response sets, due to the common underlying semantic categories in our setup (Nolden & Koch, 2022). Due to the increased number of response alternatives, we further predicted a more beneficial effect of increased preparation time for non-overlapping motor response sets than for overlapping motor response sets, possibly also a greater reduction in switch costs with increased preparation time. In addition, we predicted a smaller congruency effect for non-overlapping than for overlapping responses due to the absence of conflicting stimulus–response mappings in non-overlapping responses. Importantly, preparation time has sometimes little impact on the congruency effect (e.g., Allport et al., 1994; Huebner et al., 2004; Meiran, 1996; Rogers & Monsell, 1995), suggesting that preparation time was in these studies predominantly used to prepare for the cognitive operation required by the upcoming task and to a lesser extent to possible upcoming stimulus properties (but see Nolden et al., 2019, for preparation of distractor processing). Yet, with stimulus dimensions that have common semantic categories, it is possible that longer preparation time can help keep the two task sets separate, and this better shielding might also reduce the congruency effect.

## Methods

### Participants

Twenty-four volunteers participated in Experiment 1A (19 female, age range: 18–37 years, mean age: 25 years, all but two left-handers were right-handed). Twenty-four different volunteers participated in Experiment 1B (20 female, age range: 17–36 years, mean age: 22 years, all but two left-handers were right-handed). All participants reported normal or corrected to normal vision, normal hearing, received partial course credit for their participation and gave informed consent.

#### Sensitivity analysis

We conducted a sensitivity analysis with g*power (Faul et al., 2009) on the two most interesting effects. We chose an alpha level of 0.05 and power (1-beta) of 0.8. For the between-experiment difference in RT switch costs, we chose 0.93 as the correlation between measures (which was the correlation of repetitions and switches) and 2 as number of measurements because we conceptualized the interaction as a between-group comparison of the two transition levels. The sensitivity analysis revealed a minimum effect size f of 0.08, hence, our setup was suitable to detect even a small effect size. For the between-experiment difference in the congruency effect in the error rates, we chose 0.11 as the correlation between measures (which was the correlation of congruent and incongruent error rates), and 2 as the number of measurements, because we conceptualized the interaction as a between-group comparison of the two congruency levels. The sensitivity analysis revealed a minimum effect size f of 0.28, hence, our setup was suitable to detect a medium effect size.

### Stimuli and task

All stimuli and tasks were as in our previous paper with this paradigm (Nolden & Koch, 2022). Auditory stimuli were simple pitch tones with a duration of 500 ms, three harmonics with decreasing intensity (1/number of the harmonic), and symmetric cosine-shaped onset and offset ramps of 10 ms each. They varied in pitch (low: 300 Hz, medium: 540 Hz, high: 972 Hz) as well as loudness. Loudness varied in three levels as well (low, medium, high). The different pitch and loudness levels were combined to different combinations. Thus, while we could combine pitch and loudness orthogonally, they varied on common semantic categories ranging from low to high. We adjusted intensity individually such that participants found the maximum intensity comfortable (around 70 dB SPL for most participants). Within each of the three loudness levels, intensity was further adjusted such that low, medium, and high pitch tones had similar subjective loudness and therefore varied slightly in intensity. Sounds were created with MATLAB and were presented via Grundig VIA High Definition Audio Headphones. Visual cues were words presented centrally on a 17 inch screen with a resolution of 1280 × 1024 pixels (“TONHÖHE”, German for the pitch task, “LAUTSTÄRKE”, German for the loudness task). The cues were presented in white on black background in Courier New, font size 19 pt. Note that we used a 1:1 cue-to-task mapping, hence, differences between switch and repetition trials can be due to “real” task switch costs and cue-repetition benefits (see Logan & Bundesen, 2003; Monsell & Mizon, 2006). This 1:1 mapping was chosen because we used rather unusual tasks, compared to e.g., classifying numbers. More specifically, to render the tasks easily feasible for our participants, we decided to use concrete cues describing the tasks, i.e., a word describing the sound property that had to be classified, and any synonyms (if one wanted to achieve a 2:1 cue-task mapping) would have appeared odd and would have resulted in a confound of word frequency. Importantly, our main interest was the comparison between the two experiments and we have no reasons to assume that potential cue repetition benefits differ between them.

The visual cues instructed participants to do either the pitch or the loudness task. The auditory feature on the target dimension (pitch vs. loudness) varied in two levels (low, high), but the distractor feature varied in three levels (low, medium, high), resulting in three congruency levels: congruent (both features low or both features high), neutral (low or high target feature with medium distractor feature), or incongruent (one feature low, the other feature high).

In Experiment 1A, participants responded with “C” for low responses and “M” for high responses for both tasks (on a German standard keyboard), hence, the motor response sets overlapped for the two tasks. Participants were instructed to respond as fast and as correctly as possible. In contrast, in Experiment 1B, half of the participants responded with manual responses (“C” for low or “M” for high) to the pitch task and the other half of the participants responded with manual responses to the loudness task. For the respective other task, participants responded with vocal responses (“A” for low or “O” for high). Vocal RTs were recorded with a voice key and the response itself was noted on a form and later digitized by the experimenter.

### Procedure

**Fig. 1:**
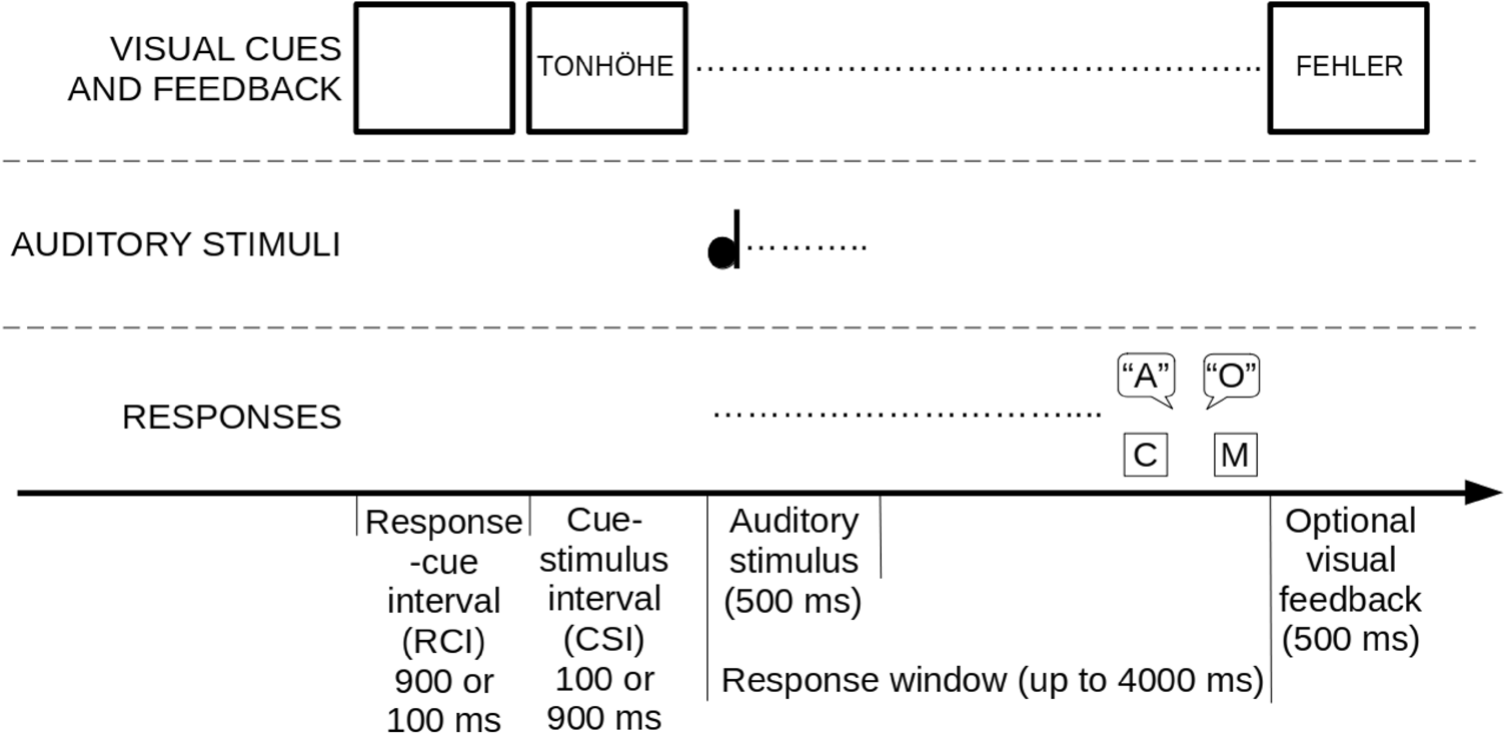
Trial procedure. Participants responded to either pitch or loudness by responding manually or vocally. A preceding cue indicated the relevant stimulus feature. Response sets were overlapping in Experiment 1A (manual responses only), and non-overlapping in Experiment 1B (manual and vocal responses). CSI (100 ms, 900 ms) and RCI (900 ms, 100 ms, respectively) were varied. We put the German words “TONHÖHE” (pitch) and “FEHLER” (error) to provide an example of the visual stimuli

The experiment lasted for around 45 min and was programmed with E-Prime 2. In total, there were 12 mixed task blocks with 96 trials each, hence 1152 trials in total. The experimental blocks were preceded by a practice block with 48 trials. The two tasks (ratio: 1:1) and all three possible congruency levels (ratio: 1:1:1) occurred in pseudo-random order. Each trial started with a blank screen which was the response-cue interval (RCI, counting from the response or feedback in trial n-1) and lasted either 900 ms or 100 ms (ratio: 1:1). The RCI was followed by the visual cue which remained on the screen until the participant responded or the end of the response window. Either 100 ms (short CSI) or 900 ms (long CSI, ratio: 1:1) after cue onset, the sound was presented for 500 ms. The response window was 4000 ms. For manual responses, there was visual feedback (“Fehler” for errors or “Schneller” for faster) in case of an error or a missing response which occurred on the screen for 500 ms. There was no feedback for correct responses. The next trial started with another blank screen (RCI). To keep the inter-trial interval constant, the RCI was varied such that the 100 ms CSI corresponded to the 900 ms RCI and the 900 ms CSI corresponded to the 100 ms RCI. The short and long CSI trials were equally distributed among the two tasks and the three congruency levels (see Fig. 1).

### Design and analysis

We compared performance with the manual responses of Experiment 1A and 1B to investigate the role of preparation time for auditory task switching and its interaction with motor response set overlap. Dependent variables were RTs and error rates.

Independent within-subject variables were transition (repetition, switch), congruency (congruent, incongruent), and CSI (short, long). Experiment (response-set overlap vs. non-overlap) served as a between-subject variable. For sake of simplicity, we did not analyze the neutral congruency level here (for further information, see Nolden & Koch, 2022).

In an additional analysis, we focused on Experiment 1B and included the vocal responses. This way we could examine the role of response modality in auditory task switching.

## Results

Practice trials and the first trial of each block (which can neither be defined as a repetition nor as a switch) were excluded from the analysis. For the RTs, trials with incorrect responses, trials with incorrect responses in the preceding trial, and RT outliers were excluded as well. RT outliers were defined as RTs < 50 ms and RTs ± 3 SD from the individual participant’s mean (~ 2% of the trials in Experiment 1A and ~ 3% of the trials in Experiment 1B, calculated separately for each response modality).

### Analysis of motor response set overlap

#### General effects independent of motor response set overlap

**Fig. 2:**
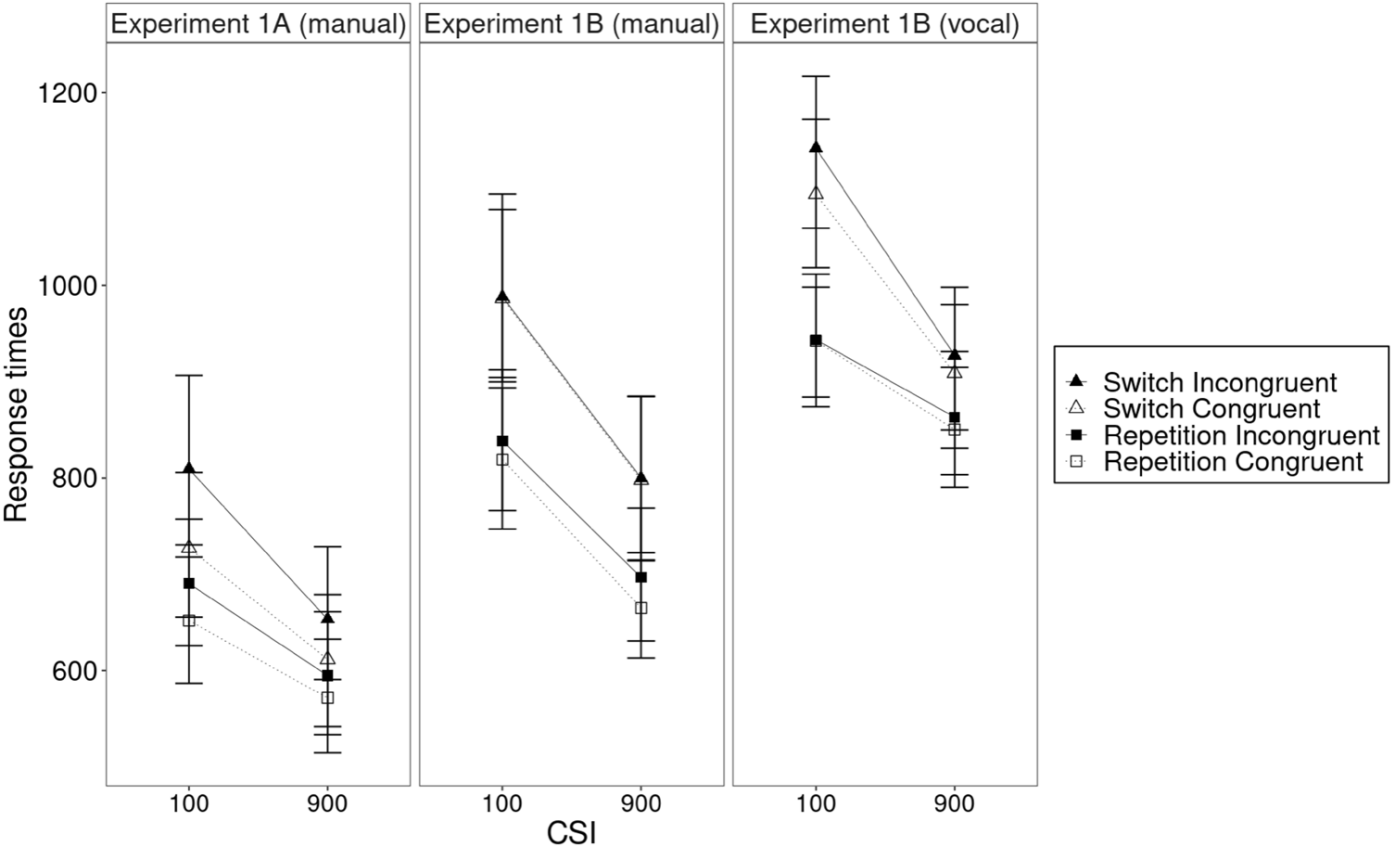
Response times in Experiment 1A (overlapping motor response sets, manual responses only) and Experiment 1B (non-overlapping motor response sets, manual and vocal responses). Error bars were calculated based on the method of Cousineau (2005) and Morey (2008)

**Fig. 3:**
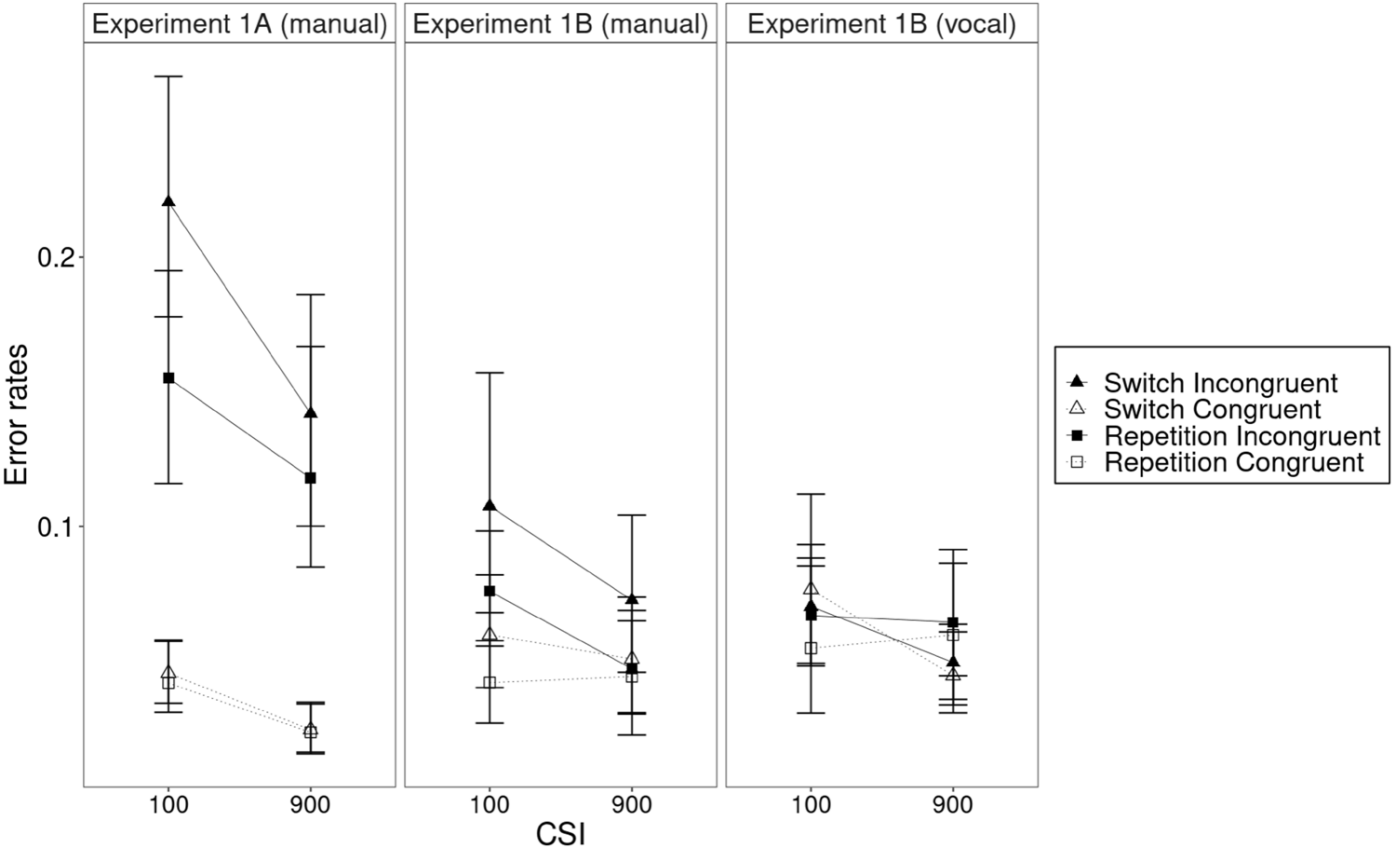
Error rates in Experiment 1A (overlapping motor response sets, manual responses only) and Experiment 1B (non-overlapping motor response sets, manual and vocal responses). Error bars were calculated based on the method of Cousineau (2005) and Morey (2008)

For the manual responses, the ANOVA with the within-subject variables transition (repetition, switch), congruency (congruent, incongruent), and CSI (short, long), and the between-subject variable experiment (response-set overlap vs. non-overlap) revealed a significant main effect of transition in the RTs, *F*(1, 46) = 87.48, *p* < 0.001, *η*_g_^2^ = 0.075, with slower responses in switches (797 ms) than in repetitions (691 ms, hence, switch costs of 106 ms, see Fig. 2). This pattern was confirmed in the error rates with a significant main effect of transition, *F*(1, 46) = 23.07, *p* < 0.001, *η*_g_^2^ = 0.023, with more errors in switches (9.9%) than in repetitions (7.7%, hence, switch costs of 2.2%, see Fig. 3).

There was also a significant main effect of congruency in the RTs, *F*(1, 46) = 16.97, *p* < 0.001, *η*_g_^2^ = 0.006, with slower responses in incongruent (759 ms) than in congruent trials (729 ms, hence a congruency effect of 30 ms), and in the error rates, *F*(1, 46) = 40.01, *p* < 0.001, *η*_g_^2^ = 0.221, with more errors in incongruent (11.7%) than in congruent trials (4.1%, hence a congruency effect of 7.6%).

The interaction of transition and congruency was significant in the error rates, *F*(1, 23) = 13.31, *p* < 0.001, *η*_g_^2^ = 0.011. The congruency effect was greater in switches (incongruent: 13.6%, congruent: 4.5%, hence a congruency effect of 9.1%) than in repetitions (incongruent: 9.9%, congruent: 3.8% ms, hence a congruency effect of 6.1%). In the RTs, the interaction of transition and congruency was further specified by experiment (see below).

In addition, there was a significant main effect of CSI in the RTs, *F*(1, 46) = 215.17, *p* < 0.001, *η*_g_^2^ = 0.124, with slower responses in the short CSI (936 ms) than in the long CSI condition (794 ms, hence a general preparation effect of 142 ms), and in the error rates, *F*(1, 46) = 40.14, *p* < 0.001, *η*_g_^2^ = 0.038, with more errors in the short CSI (9.3%) than in the long CSI condition (6.5%, hence a general preparation effect of 2.8%).

The interaction of transition and CSI was significant in the RTs *F*(1, 46) = 23.47, *p* < 0.001, *η*_g_^2^ = 0.004. In the short CSI condition, the difference between switches and repetitions was greater (878 ms vs. 750 ms, hence, switch costs of 128 ms) than in the long CSI condition (716 ms vs. 632 ms, hence, switch costs of 84 ms). The interaction of transition and CSI was significant in the errors as well, *F*(1, 46) = 4.38, *p* < 0.05, *η*_g_^2^ = 0.003. In the short CSI condition, the difference between switches and repetitions was greater (10.8 vs. 7.9%, hence, switch costs of 2.9%) than in the long CSI condition (7.2 vs. 5.8%, hence, switch costs of 1.4%). Thus, if participants had more time to prepare for the upcoming task, they could better prepare in general and also more specifically for task switches.

In addition, there was a significant interaction of congruency and CSI in the error rates, *F*(1, 23) = 23.65, *p* < 0.001, *η*_g_^2^ = 0.014. In the short CSI condition, the congruency effect was greater (incongruent: 14.0%, congruent: 4.7%, hence a congruency effect of 9.3%) than in the long CSI condition (incongruent: 9.5%, congruent: 3.6%, hence a congruency effect of 5.9%). Other studies in the literature have not found this preparatory reduction of the congruency effect (e.g., Allport et al., 1994; Huebner et al., 2004; Meiran, 1996; Rogers & Monsell, 1995), but in a recent paper we also found hints for distractor-specific preparation (Nolden et al., 2019; see also Monsell & Mizon, 2006). In the RTs, the interaction of congruency and CSI was further specified by experiment (see below).

#### Specific effects related to motor response set overlap

There was also a significant main effect of experiment in the RTs, *F*(1, 46) = 9.65, *p* < 0.01, *η*_g_^2^ = 0.156, with faster responses in Experiment 1A (overlapping motor response sets, 664 ms) than in Experiment 1B (manual responses with non-overlapping motor response sets, 824 ms). The main effect of experiment was significant in the errors as well, *F*(1, 46) = 5.59, *p* < 0.03, *η*_g_^2^ = 0.053, with more errors in Experiment 1A (overlapping motor response sets, 9.6%) than in Experiment 1B (non-overlapping motor response sets, 6.2%), suggesting a general speed-accuracy trade off.

In addition, there was a significant interaction of transition and experiment in the RTs, *F*(1, 46) = 8.25, *p* < 0.01, *η*_g_^2^ = 0.008. Switch costs were smaller in Experiment 1A (overlapping motor response sets, switches: 700 ms, repetitions: 627 ms, hence, switch costs of 73 ms) than in Experiment 1B (non-overlapping motor response sets, switches: 893 ms, repetitions: 755 ms, hence, switch costs of 138 ms).^1^ The finding that non-overlapping responses yielded greater switch costs than overlapping responses confirmed the trend observed in our previous work (Nolden & Koch, 2022). It is at odds with some of the literature that showed smaller switch costs for non-overlapping than overlapping responses (e.g., Brass et al., 2003).

There was also a significant interaction of congruency and experiment in the RTs, *F*(1, 46) = 5.00, *p* < 0.04, *η*_g_^2^ = 0.002. The congruency effect was greater in Experiment 1A (overlapping motor response sets, incongruent: 687 ms, congruent: 641 ms, hence, a congruency effect of 46 ms) than in Experiment 1B (non-overlapping motor response sets, incongruent: 831 ms, congruent: 817 ms, hence, a congruency effect of 14 ms). The interaction of congruency and experiment was significant in the error rates as well, *F*(1, 46) = 16.81, *p* < 0.001, *η*_g_^2^ = 0.107. As in the RTs, the congruency effect was greater in Experiment 1A (overlapping motor response sets, incongruent: 15.9%, congruent: 3.4%, hence, a congruency effect of 12.5%) than in Experiment 1B (non-overlapping motor response sets, incongruent: 7.6%, congruent: 4.9%, hence, a congruency effect of 2.7%).

In addition, there was a significant interaction of transition, congruency, and experiment in the RTs, *F*(1, 46) = 9.28, *p* < 0.01, *η*_g_^2^ = 0.001. To better understand the three-way interaction, we conducted two ANOVAs with the variables transition and congruency, for each experiment separately. For Experiment 1A, the interaction of transition and congruency was significant, *F*(1, 23) = 14.76, *p* < 0.001, *η*_g_^2^ = 0.028. The congruency effect was greater in switches (incongruent: 731 ms, congruent: 669 ms, hence a congruency effect of 62 ms) than in repetitions (incongruent: 643 ms, congruent: 612 ms, hence a congruency effect of 31 ms). For Experiment 1B, the interaction of transition and congruency was not significant, *F*(1, 23) = 2.19, *p* > 0.15, *η*_g_^2^ = 0.001. It thus was more difficult to ignore the irrelevant feature dimension when it has just been relevant in the previous trial than in repetitions, again particularly regarding its specific stimulus–response mappings (Experiment 1A) rather than regarding its value, i.e., high or low (Experiment 1B).

There was also a significant interaction of CSI and experiment in the RTs, *F*(1, 46) = 8.72, *p* < 0.01, *η*_g_^2^ = 0.006. Participants benefited less from preparation time in Experiment 1A (overlapping motor response sets, short CSI: 720 ms, long CSI: 608 ms, hence, general preparation benefits of 112 ms) than in Experiment 1B (non-overlapping motor response sets, short CSI: 908 ms, long CSI: 740 ms, hence, general preparation benefits of 168 ms). In the error rates, the interaction of CSI and experiment was significant as well, *F*(1, 46) = 5.61, *p* < 0.03, *η*_g_^2^ = 0.005, but pointed to the opposite direction, hence suggesting a speed-accuracy trade off. Participants benefited more from preparation time in Experiment 1A (overlapping motor response sets, short CSI: 11.6%, longs CSI: 7.7%, hence, general preparation benefits of 3.9%) than in Experiment 1B (non-overlapping response sets, short CSI: 7.1%, long CSI: 5.4%, hence, general preparation benefits of 1.7%).

In addition, the interaction of CSI, congruency, and experiment was significant in the RTs, *F*(1, 46) = 5.42, *p* < 0.03, *η*_g_^2^ = 0.001. To better understand the three-way interaction, we conducted two ANOVAs with the variables congruency and CSI, for each experiment separately. For Experiment 1A, the interaction of congruency and CSI was significant, *F*(1, 23) = 10.87, *p* < 0.01, *η*_g_^2^ = 0.022. In the short CSI condition, the congruency effect (incongruent: 750 ms, congruent: 690 ms, hence a congruency effect of 60 ms) was greater than in the long CSI condition (incongruent: 624 ms, congruent: 592 ms, hence a congruency effect of 32 ms). In contrast, for Experiment 1B, the interaction of congruency and CSI was not significant, *F* < 1. Thus, if participants had more time to prepare for the upcoming task, they could better deal with the competing irrelevant feature dimension, particularly regarding the specific stimulus–response mappings (Experiment 1A) rather than regarding its value, i.e., high or low (Experiment 1B). All other effects were not significant, all *F* < 2.14 (RTs) or *F* < 2.88 (error rates).^2^

### Analysis of response modality

To better understand the role of manual or vocal response modality, we analyzed the data of Experiment 1B with a repeated-measures ANOVA with the variables transition, congruency, CSI, and response modality (Philipp & Koch, 2011). To avoid redundancy, we only report effects containing the variable response modality (see Table 1 for the full analysis). The ANOVA revealed a significant main effect of response modality in the RTs, *F*(1, 23) = 29.76, *p* < 0.001, *η*_g_^2^ = 0.114, with faster responses in the manual task (824 ms) than in the vocal task (959 ms, see Fig. 2). The main effect of response modality was not significant in the error rates, *F* < 1 (see Fig. 3).

**Table 1:** Results of the ANOVA on the role of response modalities (Experiment 1B)

	RTs			Error rates		
	*F*	*p*	*η*_g_^2^	*F*	*p*	*η*_g_^2^
Transition	**79.17**	**0.001**	**0.104**	1.85	0.19	0.006
Congruency	**8.95**	**0.01**	**0.002**	**4.66**	**0.04**	**0.015**
CSI	**142.48**	**.001**	**0.146**	**18.34**	**0.001**	**0.015**
Response modality	**29.76**	**0.001**	**0.114**	0.03	0.87	0.000
Transition × congruency	0.01	0.93	0.000	0.26	0.62	0.000
Transition × CSI	**47.83**	**0.001**	**0.010**	**7.55**	**0.01**	**0.005**
Transition × response modality	0.86	0.36	0.001	3.79	0.06	0.008
Congruency × CSI	0.02	0.90	0.000	**4.84**	**0.04**	**0.003**
Congruency × response modality	0.15	0.70	0.000	1.52	0.23	0.008
CSI × response modality	2.51	0.13	0.001	0.50	0.49	0.000
Transition × congruency × CSI	1.59	0.22	0.000	0.70	0.41	0.001
Transition × congruency × response modality	**5.42**	**0.03**	**0.001**	1.17	0.29	0.003
Transition × CSI × response modality	**9.28**	**0.01**	**0.003**	1.22	0.28	0.001
Congruency × CSI × Response modality	0.62	0.44	0.000	**5.98**	**0.02**	**0.004**
Transition × congruency × CSI × response modality	0.43	0.52	0.000	0.18	0.68	0.000

Significant effects are printed in boldface

There was also a significant interaction of transition, CSI, and response modality in the RTs *F*(1, 23) = 9.28, *p* < 0.01, *η*_g_^2^ = 0.002. To better understand the three-way interaction, we conducted two further repeated measures ANOVAs with the variables transition and CSI, separately for the manual and the vocal response modality. While the interaction of transition and CSI was significant for both the manual responses, *F*(1, 23) = 9.56, *p* < 0.01, *η*_g_^2^ = 0.003, and the vocal responses, *F*(1, 23) = 35.07, *p* < 0.001, *η*_g_^2^ = 0.026, the preparatory reduction of switch costs was smaller in the manual task (short CSI condition: switch costs of 158 ms, long CSI condition: switch costs of 118 ms, hence, a preparatory reduction of switch costs of 40 ms) than in the vocal task (short CSI condition: switch costs of 176 ms, long CSI condition: switch costs of 61 ms, hence, a preparatory reduction of switch costs of 115 ms). Thus, the vocal response modality showed a remarkably greater preparatory reduction of switch costs than the manual response modality.

There was a significant interaction of transition, congruency, and response modality in the RTs, *F*(1, 23) = 5.42, *p* < 0.03, *η*_g_^2^ = 0.001. To better understand the three-way interaction, we conducted two further repeated measures ANOVAs with the variables transition and congruency, separately for the manual and the vocal response modality. The ANOVA did not yield a significant interaction of transition and congruency, neither in the manual, *F*(1, 23) = 2.19, *p* > 0.15, *η*_g_^2^ = 0.001, nor in the vocal response modality, *F*(1, 23) = 2.88, *p* > 0.14, *η*_g_^2^ = 0.001. Descriptively, there was a smaller congruency effect in switches (2 ms) than in repetitions (26 ms) in the manual task. In the vocal task, the pattern was inverse with a greater congruency effect in switches (33 ms) than in repetitions (7 ms). This pattern is not very conclusive and was also not observed in our previous work (Nolden & Koch, 2022).

There was a significant interaction of congruency, CSI, and response modality in the error rates, *F*(1, 23) = 5.98, *p* < 0.03, *η*_g_^2^ = 0.004. To better understand the three-way interaction, we conducted two further repeated measures ANOVAs with the variables congruency and CSI, separately for the manual and the vocal response modality. The interaction was significant in the manual response modality, *F*(1, 23) = 11.28, *p* < 0.01, *η*_g_^2^ = 0.016, with a greater congruency effect in the short CSI condition (4.1%) than in the long CSI condition (1.3%), consistent with the between-experiment analysis reported above. The interaction of CSI and congruency was not significant in the vocal response modality (*F* < 1).

## Discussion

This study examined the impact of preparation and shifting between different response sets in cued auditory task switching. In Experiment 1A, there were overlapping manual responses and in Experiment 1B, there were non-overlapping manual or vocal responses. The data revealed that increased CSI reduced switch costs, but this preparatory reduction of switch costs was similar for manual responses in both motor response sets variants. There was a smaller congruency effect for non-overlapping than for overlapping motor response sets. In contrast, switch costs were even greater for non-overlapping motor response sets than for overlapping motor response sets. Importantly, increased preparation time did not reduce switch costs more in case of non-overlapping than overlapping motor response sets.

### Preparation time and its impact on motor response set overlap and response modality

Our data revealed a greater general (not switch-specific) preparatory benefit for the non-overlapping motor response sets than for the overlapping motor response sets, when directly comparing the manual RTs of Experiment 1B (non-overlapping motor response sets) with the manual responses of Experiment 1A (overlapping motor response sets), whereas the error rates showed the opposite pattern, suggesting a speed-accuracy trade off. Note that in Experiment 1B, there was a higher number of response alternatives (as in other relevant studies such as Brass et al., 2003), such that preparation time may have been used to update working memory in this more complex setting. Importantly, increased preparation time did not reduce switch costs more in case of non-overlapping than overlapping motor response sets. This result suggests that preparation time is not particularly used to prepare to specific sets of motor responses before stimulus onset. It thus seemed as if participants, in case of non-overlapping motor response sets, prepared predominantly for aspects other than the mapping of the manual responses to the common underlying semantic categories. The irrelevant responses (irrelevant response modality) may have rather been inhibited only after selecting the correct semantic category (low or high). However, this interpretation is based on a null-effect, and further research with additional experimental manipulations and greater sample sizes are needed to better elucidate the specific mechanisms.

Experiment 1B (non-overlapping motor response sets) also revealed a greater preparatory reduction of switch costs for vocal than for manual responses while general switch costs were similar for both response modalities. This is a new response modality effect which illustrates that preparation time was used more effectively for the selection of the relevant motor response modality and/or relevant response options when participants responded vocally as opposed to manually (see also Hoffmann et al., 2022; Philipp & Koch, 2011, for a discussion of response modality effects in task switching). Note that we used a novel paradigm with auditory stimuli and tasks that shared common underlying semantic categories (Nolden & Koch, 2022), so it is yet to be shown whether this particular modality effect generalizes to other experimental setups.

### Motor response set overlap can reduce switch-costs

Our results clearly demonstrated that switch-costs are not necessarily reduced by reduced response set overlap (e.g., Brass et al., 2003; Gade & Koch, 2007; Hubner & Druey, 2006; Schuch & Koch, 2004; Yeung & Monsell, 2003). On the contrary, in our case, shifting between tasks was even more costly with reduced motor response set overlap. We assume that this was the case because our two tasks (pitch task, loudness task) share common underlying semantic categories ranging from low to high. In case of non-overlapping motor response sets (Experiment 1B), when shifting between the tasks, participants could thus not simply turn to the other task set with completely different stimulus–response mappings because stimulus properties and/or responses could be coded on the common underlying semantic categories.

Even though pitch and loudness were varied orthogonally in our paradigm, the requirement of common coding on a simple linear scale (with two possible semantic response categories, low or high) reversed the often observed benefits of non-overlapping responses. For non-overlapping responses, the identified underlying semantic category could not be mapped to a specific response, but needed to be further considered with respect to the two different motor response sets. Thus, shifting between the non-overlapping response sets of our task required an additional operation, namely shifting the response sets, and thus yielded greater switch costs than overlapping response sets. More specifically, a certain semantic response category (low or high) was mapped to two different effectors (manual, vocal), and two sets of responses (two response keys for manual responses, i.e., letters C and M on the keyboard, and two vocal-verbal responses, i.e., spoken letters A and O). This may have created additional interference which needed to be resolved after choosing the semantic response category. We thus conclude that response set overlap can impede shifting between tasks when relevant stimulus properties are substantially different, but can also reduce switch costs when the presence of common underlying semantic categories helps response selection.

Importantly, there is no general advantage of either non-overlapping or overlapping motor response sets in our paradigm. Other than the switch costs discussed above, the results also revealed a smaller congruency effect for non-overlapping than for overlapping motor response sets. We interpret this finding as stronger task interference when responses overlap than when they do not overlap (e.g., Wendt & Kiesel, 2008). More specifically, in Experiment 1A, three aspects of the tasks were respectively mapped to each other, namely stimuli, semantic categories, and physical responses, thus resulting in three possible sources of interference. In Experiment 1B, interference could only occur at the associations of stimuli and semantic categories. In this case, congruency effects were still observed, but they were much smaller compared to Experiment 1A. In summary, even though shifting came with more costs in the non-overlapping than in the overlapping setting, there was better shielding from irrelevant information for the non-overlapping motor responses than for overlapping motor responses.

### Conclusion

We investigated task switch preparation in a variant of cued auditory task switching with different levels of motor response sets overlap. Our data confirmed our previous observations that overlap in motor response sets can reduce switch costs when there are common underlying semantic categories concerning both tasks. Importantly, increased preparation time did not reduce switch costs more in case of non-overlapping than overlapping motor response sets. This result suggests that preparation time is not particularly used to prepare specific sets of motor responses before stimulus onset.

## Data Availability

Data can be made available upon request.

## Footnotes

1 Please note that in addition to the greater manual switch costs in Experiment 1B than 1A, the switch costs in the vocal response modality (switches: 1018 ms, repetitions: 900 ms, hence, switch costs of 118 ms, not included in the current analysis) were descriptively greater than in Experiment 1A as well.

2 The interaction of transition, CSI and experiment in the RTs was among the non-significant interactions, *F*(1, 46) = 0.15, *p* > .69, η_g_^2^ = .000, switch costs in Experiment 1a: 97 ms for short CSI and 50 ms for long CSI; switch costs in Experiment 1b: 158 ms for short CSI and 118 ms for long CSI. The same ANOVA was also non-significant in the error rates, *F*(1, 46) = 0.84, *p* > .36, η_g_^2^ = .001, switch costs in Experiment 1a: 3.5% for short CSI and 1.3% for long CSI; switch costs in Experiment 1b: 2.3% for short CSI and 2.2% for long CSI.
